# Prevalence and resistance to gastrointestinal parasites in goats: A review

**DOI:** 10.14202/vetworld.2022.2442-2452

**Published:** 2022-10-21

**Authors:** Takalani Judas Mpofu, Khathutshelo Agree Nephawe, Bohani Mtileni

**Affiliations:** Department of Animal Sciences, Tshwane University of Technology, Private Bag X680, Pretoria 0001, South Africa

**Keywords:** immunoglobulin heavy chain, interferon-gamma resistant, interleukin, major histocompatibility complex, resilience, strongyles

## Abstract

Gastrointestinal parasitism, particularly nematode infection, is a major health issue affecting goats worldwide, resulting in clinical diseases and productivity loss. Prevalent gastrointestinal parasites (GIPs) affecting goats in South Africa are the *Strongyloides papillosus*, *Eimeria* spp., and *Strongyles*, especially the *Haemonchus* *contortus* and *Trichostrongylus* spp. According to the issues discussed in this paper and by other authors, the prevalence and intensity of various GIPs vary with an animal’s location, breed, age, sex, and season. Because GIPs easily develop resistance to chemical treatment, selecting and breeding genetically GIP-resistant animals would be a relatively simple and inexpensive strategy for reducing or eliminating the current reliance on chemotherapy. Potential phenotypic indicators for selecting GIP-resistant goats include parasitological, immunological, and pathological phenotypic markers. Synergistic use of these indicators should be encouraged for a more accurate simplified genotype selection of resistant animals. Genes with Mendelian inheritance, particularly those involved in immunoregulatory mechanisms, have been identified in goats. Exploring this knowledge base to develop cost-effective molecular tools that facilitate enhanced genetic improvement programs is a current challenge. Future statistical and biological models should investigate genetic variations within genomic regions and different candidate genes involved in immunoregulatory mechanisms, as well as the identification of single nucleotide polymorphisms known to affect GIP infection levels.

## Introduction

Domestic goat (*Capra hircus*) rearing plays a crucial role in meeting rural households’ nutritional, social, and economic needs [1]. Notably, gastrointestinal parasitism, especially nematode infection, is a major health issue affecting the goat industry worldwide, resulting in clinical diseases and productivity loss [2–5]. Gastrointestinal parasites (GIPs) in goats are omnipresent in various environments. They may cause poor body condition [5], reduced feed intake and weight gain, and weight loss [4, 6–9], immunity [7, 9], decreased milk production and lactation period [4, 7, 9], decreased work capacity [7, 9], mortality [6, 8], abortion [7, 9], diarrhea dysentery, and anemia [10].

At present, the primary control strategy for GIPs, especially nematode infections, is the use of anthelmintic drugs [11, 12], which are associated with anthelmintic resistance [13, 14]. Bioactive forages/ethnoveterinary products [15, 16], fungi-based biological control [17, 18], grazing, and nutritional management [19, 20] are also used to control GIPs. The increased resistance of GIPs to anthelmintic drugs suggests that chemotherapy is no longer a viable option. To reduce or eliminate the current reliance on chemotherapy, alternative control measures are required. Selecting and breeding genetically nematode-resistant animals would be a relatively simple and inexpensive method of reducing the effects of nematode infestation [21].

This paper critically reviews the historical and current literature on the prevalence, intensity, and resistance of goats to GIP infection, focusing on phenotypic indicators of resistance, major genes, and quantitative trait loci (QTLs) associated with resistance to GIPs in goats.

## Common GIPs in Africa

The most prevalent GIPs affecting goats in Africa are *Strongyloides* *papillosus*, *Eimeria* spp., and *strongyles*, especially *Haemonchus contortus* and *Trichostrongylus* spp. belonging to the order Strongylida (Table-1) [6, 22–35]. Reproduction-capable adults are present in the digestive system, and fertilized females produce a fair number of eggs (70–150 mm) that are passed in the feces and hatch within 1–2 days. The only common cestode infection in small ruminants, especially goats, is *Moniezia* spp. [6, 22, 36, 37]. In the tropics, the existence of *Moniezia* spp. is linked to the ingestion of oribatid mites infected with cysticercoids of *Moniezia* spp. [6].

**Table-1:** The most common GIPs affecting goats in tropic and sub-tropics of Africa.

Species	Features	Prepatent period	Optimal temp. (°C)	Predilection site	Country Reference
*Haemonchus contortus*	Red pseudo coelomic fluid and white coiled ovaries to give barber pole appearance. It can be readily seen as thin red hair-like worms on the abomasal surface	2–4 weeks	20–25	Abomasum	Ethiopia [25], Tanzania [26], Kenya [27], Zimbabwe [28, 29], South Africa [22, 30–32], Nigeria [33], Cameroon [6]
*Trichostrongylus vitrinus*	Equal length spicules with sharp tips	2–4 weeks	20–25	Anterior small intestine	Ethiopia [24, 25, 34], South Africa [30, 32]
*Trichostrongylus axei*	Dissimilar spicules of unequal length	2–4 weeks	20–25	Abomasum	South Africa [30, 32], Ethiopia [25, 34]
*Strongyloides* spp.	A slender like worm measuring 3.5–6 mm long	9–14 days	>10, 20 opt.	Small intestine	South Africa [22, 30, 32], Ethiopia [25]
*Oesophagostomum columbianum*	Have two leaf crowns and a shallow buccal capsule. Position of cervical papillae used for species differentiation	45 days approx.	25	Large Intestine	South Africa [22, 30, 32], Ethiopia [25, 34]
*Eimeria* spp.	Parasite oocysts, a round-shaped developmental stage, are shed in profuse amounts in the feces of infected animals	7–23 days	23	Small intestine, caecum, and colon	South Africa [22, 31], Ethiopia [34], Kenya [27], Tanzania [26], Nigeria [33], Zimbabwe [28, 29]
*Monezia* spp.	The scolex and neck are tiny, but the strobila is a lengthy chain with species-specific design and sexual organs that develop at different times. The sexual organs are repeated in each proglottid and are immature, mature, and gravid, respectively, from anterior to posterior strobila	30–52 days	28	Small intestine	South Africa [22], Ethiopia [34], Egypt [35]

GIPs=Gastrointestinal parasites

## Prevalence of GIP in Goats

### Prevalence and infection intensity of important GIP in goats

The prevalence of GIP infection in livestock varies according to management practices, season, agro-ecological zone/geographical location, age, and sex of animals. The higher prevalence and intensity of strongyles, mainly *H. contortus*, could be attributed to its short generation interval and its ability to reproduce at an alarming rate if environmental conditions are favorable. The variation in the prevalence and intensity of infection by these GIPs may be explained by differences in sampling sites and size, years, genetic variations among goat breeds and agro-ecological zone conditions, and variability in management practices, such as feeding, watering, housing, rearing, stocking rate, and health control measures. The presence of subclinical rates of several GIP infections, alone or concurrently, in clinically healthy flocks may be significant for two reasons: First, infected goats can be potential carriers and may influence the extent of disease-precipitating infection in the vulnerable group of kids [38]. Second, subclinical infection with *Eimeria* spp. alone or concurrently with other GIPs could negatively affect goat’s productivity (e.g., reduced growth and milk production) [39].

### Effect of season on the prevalence and intensity of GIPs

Season has been linked to the prevalence or intensity of various GIPs [22, 24, 33, 39, 40]. The wet hot months/winter season has a higher GIP prevalence and a higher infection rate than the dry winter season [39, 40]. High humidity and temperature are desirable for the development, optimal sporulation/hatching, survival, and translocation of the preparasitic stages of GIP. Severe environmental conditions in winter force most GIPs, such as strongyles, to undergo hypobiosis. Furthermore, reduced grazing hours reduce the chances of contact between the host and parasites, resulting in a lower winter prevalence [39]. Then, worm populations dropped significantly, with the lowest percentage occurring near the peak of the dry season. However, a higher GIP prevalence has also been reported [22, 24]. The continued existence of GIPs in animals, even during the dry season when environmental conditions prevent the development and survival of their preparasitic stages, can be attributed to host animals carrying infection within them from one favorable season to the next [22].

### Effect of geographical location/agro-ecological zones on GIP prevalence

The prevalence and intensity of various GIPs vary from one agro-ecological zone/geographical location to another [22, 27, 41]. *Eimeria* spp. infection is the most prevalent GIP in goats in different regions [39, 42], resulting in coccidiosis. The agro-ecological zone factors, including temperature, rainfall, and moisture, are essential in the hatching of viable eggs, survival, and development of the parasite [22, 27, 41], leading to differences in GIP prevalence. Consequently, parasite–environment interactions influence disease transmission [43]. However, a lack of statistically significant variation in GIP prevalence among small ruminants (sheep and goat) in different regions/locations has also been observed [34].

### Effect of animal sex on GIP prevalence and intensity

Several authors have reported GIP prevalence in different goat sexes [22, 34, 39, 44–46]. Females have a higher GIP abundance than males [40, 45], which has been attributed to stress and reduced immune function during pregnancy, cycle parturition, and lactation, resulting in a decrease in the animal’s natural body resistance to parasites. However, infection occurs more frequently in males than females [22, 44, 46]. Similar prevalence and intensity between sexes have been reported in small ruminant animals [23, 34, 40], due to the same management system in which both sexes are kept, resulting in an equal chance of infection for both sexes. Inconsistencies in GIP prevalence reports in goats of different sexes are caused by various factors, including genetic variations within and among goat breeds, location in sampling sites and size, and years.

### Effect of animal age on GIP prevalence

Several authors have reported GIP prevalence in goats of different ages [22, 34, 39, 47, 48]. Young goats showed higher parasitic infections than adult goats [22, 29, 48]. Adult animals can gain parasite immunity through repeated challenges and can remove the parasite before infection occurs [37, 49]. Due to immunological immaturity and unresponsiveness [37], failure to separate young animals from adult stock at pre-weaning age, and overgrazing of infested pastures, young animals are vulnerable to infections [50]. However, some researchers have found a higher prevalence in adults than young goats [34, 47]. Dabasa *et al*. [34] and Verma *et al*. [39] observed a higher risk/prevalence of *Eimeria* spp. and strongyles in weaners (6–12 months) compared to adult goats (>12 months). However, the prevalence of GIPs [6] and *Moniezia* spp. infection [39] was similar in suckling and weaners.

## Natural Resistance to GIP Infection

Resistant animals are broadly defined as those with an increased ability to acquire and form a proper immune response to GIPs, resulting in reduced worming [51]. Sometimes, it is the result of gene modifications other than the actual drug target, particularly transporters and drug metabolism. The natural variation in susceptibility to GIPs is regulated genetically [11, 52–54] and varies between breeds and species. Variations in the crucial genes involved in the immune response are associated with resistance [55–57]. Notably, variations exist in goat breed’s ability to resist GIP infection (Table-2) [58–65]. Accumulating evidence of variability within breeds in natural immunity to GIPs has revealed that rearing animals that are less reliant on anthelmintic drenches are a viable method for controlling GIP infections [66], especially given the growing need to reduce drug use and promote organic livestock production [50].

**Table-2:** Goat breed differences in resistance to GIPs infection.

Resistant breed	Susceptible breed	Type of GIPs	Reference
Sabi	Dorper	*H. contortus*	[58]
Small East African	Galla	*H. contortus*	[59]
West African dwarf	Red Sokoto and Sahel White	*H. contortus*	[60]
Jamunapari	Barbari	*H. contortus, Strongyloides, Oesophagostomum* spp.	[61]
Creole	-	*H. contortus*	[62, 63]
Mubende	Small East African and Kigezi		[64]
Zimbabwean indigenous goats		*Eimeria*, *Strongyloides* spp.	[65]

GIPs=Gastrointestinal parasites, *H. contortus*=*Haemonchus contortus*

Animals may be bred with a high tolerance/resilience to GIPs, where they would be productive amid their worm infection intensity [67, 68]. Notably, compared to other animal species, genetic change in goat development has been slow, which cannot be attributed to the resources available to breeders and geneticist advisors. A detailed understanding of the genes and/or QTL and mechanisms involved in protective immunity would assist in the simplified genotype selection of resistant animals, which is a cost-effective way of improving productivity. This may lead to vast epidemiological benefits, accelerate genetic gain and goat productivity that is both cumulative and permanent, and application of essential principles of genomics. The genetic and physiological mechanisms underlying GIP resistance are complex and remain underexplored. Several phenotypic indicators of naturally resistant animals to GIP infection are used in selecting breeding animals.

### Phenotypic indicators of gastrointestinal nematode resistance

Several potential indicators used to evaluate resistance to GIPs include parasitological, immunological, and pathological phenotypic markers [69]. Table-3 [5, 21, 51, 54, 65, 69–80] lists some phenotypic indicators of GIP resistance.

**Table-3:** Potential phenotypic indicators of resistance to GIP in ruminants.

Phenotypic indicator	Interpretation	Reference
FEC	Lower FEC is observed in resistant animals	[21, 65, 70-72]
PCV	High PCV and low FEC is observed in resistant animals	[21, 65, 73]
Number and size of adult nematodes	Reduced number and size of adult nematodes and increased number of inhibited L_4_ are observed in resistant animals. It involves animal slaughter, consequently, it cannot be used in selecting breeding animals	[21, 74, 75]
Body weight	In the GIP-resistant goats, the greater body weight gain is attributed to their ability to survive with the infection especially in comparison with non-resistant goats	[76]
IgA	Resistant animals produce more IgA against specific parasite molecules	[5, 69, 77]
IgGI	A high level of IgGI serum level is a good indicator of responsiveness against the GIP challenge	[51]
Plasma pepsinogen	Increased plasma pepsinogen is observed in heavily infected animals and can be used to select resistant animals	[54, 69, 77]
Serum gastrin	Increased serum gastrin is observed in non-resistant animals	[78]
Eosinophil, basophil, and neutrophil	Following the GIP infection, serum antibodies to fight off the larva and adult GIP worms rise	[76, 79, 80]

GIPs=Gastrointestinal parasites, FEC=Fecal egg count, PCV=Packed cell volume, Ig=Immunoglobulin, IgGI=Immunoglobulin G index

#### Parasitological phenotypic markers

Parasitological phenotypic markers include worm burden, fecal egg count (FEC), fecundity, and worm length [69]. The FEC measured as egg per gram (EPG) [21, 70–72] and packed cell volume (PCV) [21, 65, 73, 76] are the most commonly used phenotypic indicators of host resistance. Fecal egg count, a secondary measure of the host’s worm burden in the stomach [21], can indicate the degree of nematode infection and provide a direct estimate of pasture contamination [21, 81]. Among many phenotypic indicators, FEC is by far the most accurate, relatively easy to measure, functional, and often used predictor for assessing the possibility of host resistance and susceptibility to GIPs [21, 81]. The current merit for measuring individual animal FEC is the adapted McMaster method, with 50 EPG feces sensitivity [82]. A moderate to high (r ~ 0.7) correlation exists between FEC and worm burden. However, this varies with the GIP species, host breed investigated [52, 59, 62, 63], inhibition of infective larvae, and suppression of worm fecundity [83]. The FEC heritability ranges between 0.14 and 0.40, depending on the GIP species and breed surveyed [62, 84]. The FEC of goats on extensive grazing ranges from nearly zero to several thousand in some individuals [29], with a threshold value of 2000 EPG of feces indicating a heavily parasitized animal [85].

Feces from goats infected with GIPs contaminate the ecosystem and are consumed by the rest of the flock, increasing the parasite’s total population [86]. Resistant goats have approximately 50% lower FECs [70, 71, 76], a lower nematode burden, reduced egg laying, and decreased EPG in feces than susceptible goats. Selecting resistant breeding stock using FEC requires a relatively high GIP challenge to accurately assess their phenotype, which may lead to lower production when withholding the drench [51]. It is also expensive to calculate in a commercial farming setting, and due to physiological complexities, it cannot reflect all paths involved in GIP resistance [69]. Selection based on low FEC is feasible in the medium to long term, as GIPs are slow to adapt to resistant hosts [81].

#### Immunological phenotypic markers

Immunological phenotypic markers include antibody responses and levels of different antibodies (immunoglobulin [Ig]A, IgG, IgE, and IgM) and blood eosinophils [55, 69, 77]. Immunoglobulin A levels in the serum are positively correlated with other immune parameters (eosinophils, mast cells, and globule leucocytes), whereas they are negatively correlated with GIP worm length and FEC [87]. Immunoglobulin A plasma has high heritability and repeatability [55] and eosinophil plasma levels [88]. IgG serum levels have also been suggested to be a good indicator of responsiveness against the L3 of GIP and could possibly be used to select resistant animals [51]. A primary role of eosinophils in killing larva different from GIPs has been reported [89]. McBean *et al*. [90] believed that the use of eosinophilia as a predictor of the response to GIP infection in goats is probably of minimal value due to its weak correlation with FEC.

#### Pathological phenotypic markers

Pathological phenotypic markers include PCV, plasma pepsinogen, and live weight [69, 77]. PCV is the percentage of red blood cells in the blood and is usually above 30% in goats [91]. PCV could be used as a valuable indicator of blood-sucking parasites [21, 73] and to determine whether livestock breeds are resistant to GIPs [70, 72, 76]. When PCV drops below 20%, anemia develops [91] as a clinical sign of parasite infection. In essence, resistant animals exhibit high PCV and low FEC; low PCV values are attributed to a high FEC, which is attributed to the adult parasite sucking a substantial amount of blood from the abomasum [21, 92, 93]. A significantly strong negative relationship exists between FEC and PCV [94–96]. Plasma pepsinogen is a pathophysiological indicator of abomasal lesions induced by the size of the GIP worms [54], which are formed by the abomasal chief cells and converted from pepsin by hydrochloric acid. The development and emergence of the GIP larva (L4) in the abomasum leads to the loss of parietal cells of the gastric glands, resulting in reduced synthesis of hypochloric acid. The heritability of the serum levels of pepsinogen ranges from low to moderate, with a value of 0.21 [54].

Notably, based on the literature on several phenotypic indicators of resistance in animals discussed above, phenotypic should not be used in isolation as diagnostic tools for GIP infection; however, a combination of these phenotypic indicators could result in a more accurate simplified genotype selection.

## Genetic Markers of GIP Resistance

Mapping and characterizing the QTLs and/or genes involved in various biological processes are important in studying GIP resistance complexity. Studies have been conducted on the genetic markers for GIP resistance in goat breeds and GIP species. As a result, several QTLs and genes have been identified. Different GIP species may not be susceptible to the same immune responses. Therefore, mapping QTLs influencing resistance to specific GIP species requires an extensive phenotypic study of a population structure suitable for statistical analysis. Genes that potentially affect immune responses are associated with metabolism, mitochondrial function, longevity, heat shock responses, and other physiological responses [97]. Several studies have been conducted to identify QTLs for GIP resistance in ruminants (Table-4) [2, 11, 97–115]. The mechanisms underlying GIP resistance have been studied [53]. The difficulty in classifying the various genes associated with GIP resistance stems from the fact that many genes have been linked to this trait. Several studies have reported the genes and/or putative QTLs responsible for resistance to GIP infection; lack of consensus among studies may be attributed to the complexity of this trait [116, 117].

**Table-4:** Candidate genes for resistance to GIPs.

Candidate genes	Interpretation	Reference
MHC class II locus DRB1	The MHC Class II determines antigen recognition and animals that are heterozygous have lower FEC than homozygotes	[97-101]
IFN-γ	The IFN-γ affects the detection of antigens but its main function is to identify the type of cytokine reaction. One of the two or three alleles at the IFN-γ locus is associated with high GIPs FEC	[11, 100, 102, 103]
Cytokines IL-4	The host relies almost entirely on T lymphocytes, in particular, T helper 2 (Th2) cells to eliminate GIPs during infection, which triggers the development of the specific cytokines	[104, 105, 106]
IL-2		[105, 106, 107]
IL-13		[2, 108, 109]
VbetaT-cell receptor		[110]
IgHA gene	The difference in the hinge segment of IgHA can render the molecule more or less versatile and thus more or less capable of binding antigens with a number of epitope separations of any particular pathogen	[111, 112]
TLRs (TLR-2, TLR-4 and TLR-9)	Following GIPs infection, the TLR genes are rather commonly displayed in the intestinal mucosa of genetically resistant animals	[113, 114]
The CLRs	The CLRs genes are responsible for the natural identification of carbohydrate surface found on the GIPs. The mannose receptor to bind to *Trichuris* *muris’s* excretory/secretory	[115]

GIPs=Gastrointestinal parasites, MHC=Major histo-compatibility complex, FEC=Fecal egg count, IFN-γ=Interferon gamma, IgHA=Immunoglobulin heavy chain, IL=Interleukin, CLR=C-type lectin receptors, TLR=Toll-like receptors

The most conspicuous genes for resistance to GIPs include the major histocompatibility complex (MHC) Class II locus DRB1 and interferon-gamma (IFN-γ) [97], the cytokines interleukin 4 (IL-4) [104], IL-2 and IL-13 [105–107], and the VbetaT-cell receptor (TCRVb) [110]. Such markers are candidate genes with biological plausibility.

### Major histocompatibility complex

MHC is a conspicuous gene deemed significant for disease resistance, autoimmunity, immune responsiveness, and reproductive success [118]. Caprine MHC (Cahi/GoLA/CLA) is a cell surface molecule encoded on chromosome 23 [119] by a broad gene family and implicated in antigen presentation by immune cell glycoprotein receptors [120]. MHC Classes I, II, and III are the three main groups of MHC. Heterodimeric peptide-binding proteins are encoded in MHC Classes I and II. Major histocompatibility complex Class III codes specific immune system compartments, such as elements, cytokines, and heat shock proteins. Major histocompatibility complex I in goats is 1077 bp long, encoding a specific protein with 337 amino acids. Major histocompatibility complex Class II molecules trigger an immune response in the event of an extracellular infection of interest. The DYA gene is one of the MHC Class II genes and can be partitioned into DQ and DR molecules; hence, it plays an essential role in the expansion of immune responses controlled by MHC. The association between MHC and resistance to GIP infection has been reported in different studies [69, 98–101].

The MHC Class II gene is closely linked to the microsatellite DYMS1, a possible candidate gene for resistance to *H. contortus* [121]. Among ruminants, DRB is the MHC gene complex’s most polymorphic locus [69], with a strong correlation with GIP resistance [110, 122]. Ovine DRB1 gene polymorphisms and the FECs of GIPs are significantly associated [97, 98]. DRB1*1101 gene/allelic expression is higher in meat, young, and male goats than in milch, older, and female goat breeds exposed to *H. contortus* [109]. DRB1*1101 is strongly associated with susceptibility to GIPs, especially *H. contortus*, due to the negative correlation between its expression and PCV [122].

### Cytokine genes

Cytokines are cellular-signaling proteins involved in intracellular communication that play a significant role in the immune system. The infected animals depend on T lymphocytes, especially T helper 2 (Th2) cells, to expel GIPs during infection [123, 124]. The immune response of type Th2 stimulates the synthesis of various cytokines, such as IL-4, IL-5, IL-10, IL-13, IL-25, and IL-31 [120] and IFN-γ. Furthermore, it contributes to B-cell differentiation by responding to antibody production, including IgE, IgG1, IgG4, and IgA [125], and gathers eosinophils to attack and wipe out GIPs [126]. The immune response of type Th2 can lower the immune response-mediated pathological inflammatory responses of type T helper 1 by cross repression and further challenges to the GIP lifetime [127].

Interleukins are candidate genes considered essential for immune response, resistance to diseases, autoimmunity, and reproductive efficiency. There are three main groups of the IL gene family: IL-2, IL-13, and IL-4. These functional candidate genes (IL-2, IL-13, and IL-4) are associated with resistance to several GIP species [105, 106]. Interleukin 13 genes are involved in the resistance to GIPs [2, 108, 109] and mucosal infections [128]. In goats, the role of IL-13 in the immune response to GIP infection is well documented [109, 129, 130]. IL-13 modifies the role of intestinal epithelial cells by inducing an abnormal increase in the number of goblet cells [131] and hypercontraction of smooth muscles [129]. On infection with GIPs, cells recirculating in afferent and efferent lymph reliably express the IL-13 gene in sheep [132]. During GIP infection, Th2 cells produce IL-13, which induces epithelial cell repair and thus promotes the contraction and expulsion of parasitized epithelial cells, as well as mucus development, preventing GIP contact with the epithelial surface and hastening GIP expulsion [109, 130, 133]. IL-13 and IL-4 act collectively in activating macrophages that produce metabolic products that attack and stress the larval stage of GIPs within the intestinal mucosa [134]. Male goats express more intestinal IL-13 than females, suggesting that IL-13 cytokine development in response to GIP resistance is more complex regarding animal sex [109].

Interferon-gamma is a cytokine involved in the host’s response after an immune challenge by pathogen infection [135], revealing that it is a plausible practical candidate gene for GIP resistance [136]. Interferon-gamma triggers macrophages and detects, engulfs, and destroys pathogens [135]. A polymorphism in the region near IFN-γ has been linked to increased parasite-specific plasma IgA in sheep and reduced FECs [102]. Plasma IgA production results due to responses to external peptide molecules, such as those derived from GIPs [135]. Hence, polymorphism resulting in the differentiated expression or receptor-binding affinity of IFN-γ may affect extracellular parasite resistance.

### Immunoglobulin heavy chain (IgHA) gene

Immunoglobulin A is an antibody that plays an essential role in mucosal immunity, primarily acting as a primary defense mechanism in preserving intestinal mucosa integrity and serum [137] and conferring protection against antigens that may cause epithelial wall breakdown [137]. In goats, on chromosome 20 is the QTL aligned with the unique IgA feature against GIPs [124]. The hinge region variation of IgHA may cause the molecule to be flexible [111] and thus able or unable to bind pathogens with several epitope separations of any single parasite [137], resulting in functionally and structurally different IgA molecules and dissimilarity in the IgA response to parasitism [101, 111, 112]. Polymorphism in the IgHA region influences the immune system’s response to pathogens and the consequences of infection in goats [101].

### Pattern recognition receptors (PRRs)

The germline-encoded PRRs, including the C-type lectin receptors (CLRs) [115] and toll-like receptors (TLRs) [114], NOD-like receptors, and RIG I-like receptors, are some of the first pathogen detection systems. The PRR proteins identify damage-associated molecular patterns and pathogen-associated molecular patterns. Following the GIP challenge, TLR genes (TLR-2, TLR-4, and TLR-9) are more profusely expressed in the gut mucosa of resistant animals [113]. CLRs are also eligible genes for primitive surface carbohydrate identification found in GIPs.

## Prospects and Opportunities

In the near future, genomic methods can be viewed as an effective means of controlling GIP infections. Notably, the literature on genes and/or QTL detection for GIP resistance in goats is not as extensive as that on the same subject concerning sheep. The sheep genome can be used as a blueprint due to the high level of similarity between the genomes of goats and sheep compared to other livestock species. Future studies, as well as statistical and biological models, should focus on genetic variations in genomic regions and various candidate genes involved in immunoregulatory mechanisms, as well as on the identification of single nucleotide polymorphisms known to affect GIP infection levels.

## Conclusion

This study reveals that GIPs have a negative impact on goat health and productivity. Because the effectiveness of various existing methods of controlling GIP infection in goats varies and anthelmintic resistance is likelier, a more effective method of GIP infection treatment based on genetic selection is urgently needed. There is a well-defined seasonal pattern of GIP infections in goats in different tropic and subtropic regions in Africa. The season, age, and sex of goats influence the transmission, prevalence, and intensity of GIP; however, this differed across regions, years, and GIPs investigated. The parasitological, immunological, and pathological phenotypic markers for natural resistance to GIPs should not be used in isolation, but synergizing these indicators could result in a more accurate simplified genotype selection of resistant animals. The most conspicuous genes for resistance to GIPs include the MHC, IFN-γ, ILs, TLRs, and TCRVb. Given the reviewed literature, the genetic architecture of resistance to GIP infection is a trait determined by several loci, with slight effects. The long-term consequences of GIPs are still poorly defined, and several unresolved issues exist: (i) To what degree is GIP infection capable of manipulating the immune function directly, and with what implications can it impact future infections? (ii) How does early GIP exposure impact the developing immune system? (iii) How do GIP coinfections modify host (goat) susceptibility, parasite intensity, and distribution pattern? (iv) How and which type of anemia does GIP infection cause in goats of different ages and sex?

## Authors’ Contributions

This paper is the component of the Ph.D. thesis of the first author TJM, under the guidance of KAN and BM. TJM: Conceived, framed the main ideas and prepared the first and wrote the manuscript. KAN and BM: Read, criticized, and corrected the manuscript. All authors have read and approved the final manuscript.
